# Frequency and Predictors of Radial Artery Occlusion in Patients Undergoing Percutaneous Coronary Intervention

**DOI:** 10.7759/cureus.25505

**Published:** 2022-05-30

**Authors:** Ussama Munir, Rozi Khan, Nouman Nazeer, Junaid Akhter, Anwaar ul Hassan, Bashir Hanif

**Affiliations:** 1 Cardiology, Bahawal Victoria Hospital, Bahawalpur, PAK; 2 Internal Medicine, Bolan University of Medical and Health Sciences, Quetta, PAK; 3 Internal Medicine/Hospital Medicine, Medical University of South Carolina, Florence, USA; 4 Cardiology, Tabba Heart Institute, Karachi, PAK; 5 Cardiology, Quaid-E-Azam Medical College, Bahawalpur, PAK

**Keywords:** : acute coronary syndrome, trans femoral access, trans radial access (tra), radial artery occlusion (rao), primary percutaneous coronary intervention (pci)

## Abstract

Background and objective

Transradial access (TRA) has become the preferred route for percutaneous coronary interventions (PCI), and this site is often a chink in the armor for staged PCI. In this study, we aimed to evaluate the incidence and predictors of radial artery occlusion (RAO) after TRA.

Methods

We conducted a retrospective study involving 1,307 patients who underwent PCI at the Tabba Heart Institute (THI) in Karachi, Pakistan from August 2018 to June 2019. TR band was used for hemostasis after PCI.

Results

The primary outcome of our study was RAO, which was observed in 11.3% of the study subjects. On multivariate analysis, female gender [odds ratio (OR): 1.79, 95% CI: 1.21-2.64], cardiovascular instability (OR: 2.5, 95% CI: 1.22-5.11), dyslipidemia (OR: 0.61, 95% CI: 0.4-0.92), and a higher number of diseased vessels were found to be predictors of RAO (p=0.004).

Conclusion

RAO is often an asymptomatic complication of TRA. To ensure radial artery patency, a carefully thought-out management plan and follow-up must be devised for high-risk patients.

## Introduction

Transradial access (TRA) has become the preferred route for performing coronary arteriography as well as percutaneous coronary interventions (PCI) over the last 10-20 years in the United Kingdom, Canada, and Asia but is still lagging behind transfemoral access in the United States. Over 20% of interventional cardiology procedures are performed through TRA [1]. The transradial (TR) approach was first used in 1989 for PCI [2]. TRA is advantageous over transfemoral access in reducing bleeding complications, ensuring early mobility, and improving comfort and cost-effectiveness [3]. TRA has also been associated with a reduced complication rate in high-risk patients [4]. Radial artery spasm is the most frequent complication of TR cardiac catheterization. It causes patient discomfort and reduces the procedure's success rate [5-7]. Radial artery occlusion (RAO) is an infrequent complication of TR coronary procedures. In multivariate studies, the predictors of RAO include the diameter of the sheath and its relationship to the size and diameter of the radial artery, prolonged cannulation time, number of catheter changes, radial artery spasm, body mass index, patient gender, post-procedural compression time, the presence of anterograde flow in the artery during hemostasis (patent hemostasis), and the use and dosage of anticoagulation during the procedure [8,9].

RAO is usually asymptomatic due to the dual blood supply to the hands, and hence it is often overlooked. More than 50% of the operators do not even assess radial artery patency in patients undergoing TR invasive procedures [10]. The underlying pathophysiology of RAO in the early phase involves the presence of radial artery thrombus caused by endothelial injury to the radial artery and a decrease in blood flow after cannulation. Repeated catheter propagation and manipulation may create an environment prone to thrombus formation leading to RAO [11]. Many observations about RAO provide indirect evidence to support this hypothesis of radial artery thrombus formation. RAO tends to occur early after catheterization through TRA. Approximately 50% of patients undergoing invasive procedures through TRA undergo spontaneous recanalization of the radial artery within one to three months [12]. Direct endorsement of the thrombotic hypothesis has come from recent studies that have confirmed the presence of radial artery thrombus on vascular ultrasound [13]. TR cannulation can also negatively affect and remodel radial artery structure and function. A study using optical coherence tomography found that 67% of patients had intimal tears in the cannulated radial artery and 36% had medial dissections immediately after TR PCI [14]. Other studies have found that lumen diameter and lumen area are smaller in patients undergoing repeat TR cannulation than in those who undergo first-time TR as the result of an increase in intimal hyperplasia and intima-media thickness [15]. A study of radial artery function after radial cannulation found that flow-mediated dilatation was blunted and remained blunted even nine weeks after TRA when compared with a non-cannulated radial artery. The radial artery response to nitroglycerin was also decreased and blunted, suggesting that the impairment in function was more than just temporary damage to the endothelium and involved long-term changes to the smooth muscle layer of the radial artery [16]. The incidence of RAO varies from 1.5 to 30.5%, with an average of 5-12% of patients undergoing TRA [17]. Once the radial artery is occluded, it cannot be used as an access site for future catheterizations or as an arterial conduit for bypass surgery. RAO also renders the ipsilateral ulnar artery unusable due to the risk of hand ischemia [12,16].

Due to the advances in interventional cardiology, more and more procedures are being performed through the radial approach and we need its patency for staged procedures. In light of this, our study aims to evaluate the frequency and predictors of RAO in patients undergoing PCI through radial access before discharge from the hospital.

## Materials and methods

Study design and Setting

We conducted a cross-sectional retrospective study to evaluate the frequency and predictors of RAO in patients who underwent PCI through TRA at the Tabba Heart Institute (THI) in Karachi, Pakistan. Data were collected retrospectively from August 2018 to June 2019, from medical records at the hospital.

Patient selection

Both male and female patients aged 18 years and above who underwent PCI for either acute coronary syndrome (ACS) or severe ischemic heart disease (SIHD) through TR approach using 6-French sheath where TR band was used as hemostasis device were included. Patients having radial artery fistula, abnormal Allen’s test, abnormal Barbeau test, and those who underwent repeat procedures during the same admission through the TR approach were not included.

Data collection

After obtaining approval from the Institutional Review Board at THI, data was retrieved from the institutional database. Baseline and clinical characteristics along with procedure details were retrieved. The primary outcome of RAO was defined as an absence of radial pulse using the reverse Barbeau test before discharge or 24 hours post-procedure. Secondary outcomes were predictors of RAO.

Statistical analysis

Data were analyzed using Stata Version 12. The Shapiro-Wilk test was applied to check the normal distribution of variables and was expressed as means ± SD or median (IQR) depending on whether normally distributed or not, respectively. Unpaired t-test or Mann-Whitney U test was applied accordingly to assess significant differences. The primary outcome was presented in percentages. The univariate assessment was performed by applying an appropriate chi-square test or Fisher's exact test and risk ratios with 95% CIs were reported. Multivariate analysis of predictors was performed by applying logistic regression. A two-sided p-value <0.05 was considered statistically significant.

## Results

During the study period from August 2018 to June 2019, 1,451 PCIs were performed on 1,350 patients (including 101 repeat procedures). The mean age of the patients was 57.6 ± 11.4 years; 1,025 (78.4%) of them were males and 282 (21.6%) were females. Among them, the primary outcome was unknown in 47 (3.2%) patients, and hence data of 1,307 patients were available for the final analysis. The baseline and periprocedural characteristics of the study population are depicted in Table 1. Multiple regression analysis was performed to examine the predictors of RAO after TR catheterization, and female gender, cardiovascular instability, dyslipidemia, and higher number of diseased vessels were found to predict RAO (Table 3).

**Table 1:** Baseline and periprocedural characteristics of the study population SD: standard deviation; HbA1c: glycated hemoglobin; PCI: percutaneous coronary intervention; LVEF: left ventricular ejection fraction; CABG: coronary artery bypass graft; RAO: radial artery occlusion

Variable		Value
Age, years, mean ± SD		57.6 ± 11.4
Gender, n (%)	Male	1,025 (78.4)
	Female	282 (21.6)
Initial creatinine, mg/dl, mean ± SD		1.05 ± 0.37
Peak creatinine, mg/dl, mean ± SD		1.21 ± 0.55
HbA1c, %		7.2
Fluoroscopy time, minutes, mean ± SD		24.2 ± 36.9
Contrast volume, ml, mean ± SD		180 ± 91.8
Pre-PCI LVEF, %, mean ± SD		43.6 ± 10.1
Risk factor, n (%)	Diabetes mellitus	545 (41.7)
	Dyslipidaemia	450 (34.4)
	Hypertension	785 (60.1)
	Family history	280 (21.4)
	Prior MI	200 (15.3)
Prior PCI, n (%)		186 (14.2)
Prior CABG, n (%)		34 (2.6)
Heart failure, n (%)		110 (8.4)
Dominance, n (%)	Right	1,109 (84.9)
	Left	112 (8.6)
	Co-dominance	72 (5.5)
No. of diseased vessels, n (%)	Single	525 (40.2)
	Double	480 (36.7)
	Triple	302 (23.1)
RAO, n (%)		148 (11.3)
Radial hematoma, n (%)		30 (2.3)

The frequency of female gender, hypertension, diabetes mellitus (DM), family history, prior myocardial infarction (MI), prior PCI (single and multivessel), and cardiovascular instability were calculated; the results are presented in Table 2, along with distribution of patients between RAO and non-RAO groups. The number of females in the RAO group was 47 (31.8%) and that in the non-RAO group was 235 (20.3%), as seen in Table 2. Also, in our study, DM was present in 60 (40.5%) in the RAO group and 485 (41.8%) in the non-RAO group. A cardiovascular instability was noted in 12 (8.1%) in the RAO group and 34 (2.9%) in the non-RAO group, as presented in Table 2. Significantly, a family history of MI was present in 33 (22.3%) in the RAO group and 247 (21.3%) in the non-RAO group. It is essential to note that in our study, 21 (14.2%) patients had a history of prior MI in the RAO group and 179 (15.45) had it in the non-RAO group, as shown in Table 2. The primary outcome of our study was documented in 148 (11.3%) patients. RAO was documented more commonly in patients who had cardiovascular instability during the procedure and in females 8.1% vs. 2.9% (p=0.004) and 31.8% vs. 20.3% (p=0.002), respectively (Table 2).

**Table 2:** Clinical characteristics and procedural data MI: myocardial infarction; PCI: percutaneous coronary intervention; LVEF: left ventricular ejection fraction

Variable	RAO group (n=148, 11.3%)	Non-RAO group (n=1,159, 88.7%)	P-value
Female gender	47 (31.8%)	235 (20.3%)	0.002
Hypertension	93 (62.8%)	692 (59.7%)	0.477
Diabetes mellitus	60 (40.5%)	485 (41.8%)	0.791
Family history	33 (22.3%)	247 (21.3%)	0.751
Prior MI	21 (14.2%)	179 (15.45)	0.808
Prior PCI	17 (11.5%)	169 (14.6%)	0.381
Multivessel PCI	59 (39.9%)	388 (33.5%)	0.141
Cardiovascular instability	12 (8.1%)	34 (2.9%)	0.004
LVEF	74 (53.2%)	509 (44.5%)	0.058

The frequencies of RAO were calculated with regard to the female gender, patients with dyslipidemia and cardiovascular instability, no. of diseased vessels (overall), two-vessel coronary artery disease (2VCAD) [compared to single-vessel coronary artery disease (SVCAD)], three-vessel coronary artery disease (3VCAD) (compared to SVCAD), and left ventricular (LV) dysfunction [ejection fraction (EF) 40% or less]. The results are presented in Table 3.

**Table 3:** Multiple regression analysis for predicting radial artery occlusion OR: odds ratio; CI: confidence interval; 2VCAD: two-vessel coronary artery disease; 3VCAD: three-vessel coronary artery disease; LV dysfunction: left ventricular dysfunction

Variable	P-value	OR	95% CI for OR	
			Lower	Upper
Female gender	0.003	1.794	1.215	2.649
Dyslipidaemia	0.021	0.616	0.409	0.929
Cardiovascular instability	0.012	2.506	1.228	5.115
No. of diseased vessels (overall)	0.004			
2VCAD (compared to SVCAD)	0.012	1.748	1.13	2.702
3VCAD (compared to SVCAD)	0.002	2.13	1.333	3.405
LV dysfunction (EF 40% or less)	0.169	1.291	0.897	1.858

## Discussion

The main finding of our study was that out of the total TR cardiac catheterization procedures, 11.3% of patients had RAO diagnosed by the bedside at discharge or 24 hours post-procedure as revealed by the reverse Barbeau test. The major predictors of RAO were female sex, dyslipidemia, cardiovascular instability, and a higher number of diseased coronaries, which increased the odds of having RAO.

Spontaneous recanalization of RAO with the passage of time has been documented in some studies. The incidence of RAO varied from <1 to 33% depending upon the time and choice of assessment modality as documented in a systematic review, which involved 66 studies with 31,345 patients; the documented incidence of RAO within 24 hours was 7.7%, which decreased to 5.5% on follow-up [18]. A prospective non-randomized single-center study from India that included 1,945 consecutive patients undergoing TR catheterization revealed RAO in 17.4% at 24 hours post-procedure, while the current study revealed a rate of 11.3% in a total of 1,307 patients [19]. A prospective study from Egypt documented RAO in 32.9% out of a total of 164 patients post-procedure at 24 hours, and this number was found reduced to 29.9% at the six-month follow-up examination with ultrasound Doppler [20]. Pancholy in 2009 studied the effect of HemoBand versus TR band for hemostasis after TR catheterization and showed an occlusion rate of 4.4% in the TR band group versus 11.2% in the HemoBand group, which is contrary to our findings where we used TR band and documented an RAO rate of 11.3% [21]. Dharma et al. [22] documented RAO in 11.7% of the total of 1,706 patients, which is comparable to the findings of our study.

Multivariate analysis showed that female gender, cardiovascular instability, multivessel disease, and dyslipidemia were risk factors for RAO. Similar to the findings of our study where 16.7% of females vs. 9.9% of males developed RAO (p=0.003), Sadaka et al. found female gender to be an independent risk factor for RAO (p<0.001) [20]; however, in contrast to their findings, age was not predictive of RAO in our study. Sinha et al. also documented female gender as a risk factor along with DM while our study negates this finding, as 11% of diabetics vs. 11.5% of non-diabetics developed RAO as per our findings (p=0.791) [19]. Age was not predictive of RAO in our study while some studies did document this finding [18].

An interesting fact about RAO is the way it is diagnosed, either with the bedside test along with pulse oximetry and plethysmography or the ultrasound Doppler examination, and the time at which RAO is diagnosed. The findings documented by Sinha et al. [19] are pertinent here, where a palpable pulse was present in 34% of patients diagnosed with RAO by US Doppler examination, revealing a lot of disparity in the existing data. In our study, which was retrospective in its design, unfortunately, we did not have the US doppler data available pre- and post-procedure, which would have helped us to better understand and further stratify the predictors of RAO. The pre-procedure US Doppler data of the radial artery is important in understanding its anatomy, which could aid significantly in ensuring the safety of the procedure, avoiding possible complications, and providing patient comfort [23].

Limitations

Our study has a few limitations. Primarily, it was a single-center study, with relatively small sample size. This means that the results gathered from our study cannot readily be generalized. Further studies with larger sample sizes are therefore required to truly establish the validity of certain parameters as predictors of RAO in certain individuals and patients with risk factors. Furthermore, unfortunately, we did not have the ultrasound Doppler data available pre and post-procedure, which would have led us to better understand and further stratify the predictors of RAO.

## Conclusions

In summary, the radial access for coronary procedures appears to be a safe substitute for femoral access. In addition, radial access virtually eliminates local vascular complications. However, radial access is a comparatively difficult approach, requires expertise, and may lead to a major serious outcome in the form of RAO due to thrombosis. Our study revealed an RAO incidence rate of 11.3% after TR cardiac catheterization, which could be a chink in the armor for staged procedures in certain cases. To keep the access site patent is vital in this era because of the advent of several advancements to treat heart diseases in interventional cardiology. For a better understanding of the predictors of RAO, we need registry data and should conduct more randomized control trials, which could provide insights into eliminating this potential weakness.
